# High quality permanent draft genome sequence of *Phaseolibacter flectens* ATCC 12775^T^, a plant pathogen of French bean pods

**DOI:** 10.1186/s40793-015-0127-5

**Published:** 2016-01-13

**Authors:** Yana Aizenberg-Gershtein, Ido Izhaki, Alla Lapidus, Alex Copeland, TBK Reddy, Marcel Huntemann, Manoj Pillay, Victor Markowitz, Markus Göker, Tanja Woyke, Hans-Peter Klenk, Nikos C. Kyrpides, Malka Halpern

**Affiliations:** Department of Evolutionary and Environmental Biology, Faculty of Natural Sciences, University of Haifa, Haifa, Israel; Centre for Algorithmic Biotechnology, St. Petersburg State University, St. Petersburg, Russia; Algorithmic Biology Laboratory, St. Petersburg Academic University, St. Petersburg, Russia; Department of Energy Joint Genome Institute, Genome Biology Program, Walnut Creek, CA USA; Biological Data Management and Technology Center, Lawrence Berkeley National Laboratory, Berkeley, CA USA; Leibniz Institute DSMZ—German Collection of Microorganisms and Cell Cultures, Braunschweig, Germany; School of Biology, Newcastle University, Newcastle upon Tyne, UK; Department of Biological Sciences, Faculty of Science, King Abdulaziz University, Jeddah, Saudi Arabia; Department of Biology and Environment, Faculty of Natural Sciences, University of Haifa, Oranim, Kiryat Tivon, Israel

**Keywords:** *Phaseolibacter flectens*, *Enterobacteriaceae*, plant pathogen, French bean pod, *Phaseolus vulgaris*

## Abstract

*Phaseolibacter flectens* strain ATCC 12775^T^ (Halpern et al., *Int J Syst Evol Microbiol* 63:268–273, 2013) is a Gram-negative, rod shaped, motile, aerobic, chemoorganotroph bacterium. *Ph. flectens* is as a plant-pathogenic bacterium on pods of French bean and was first identified by Johnson (1956) as *Pseudomonas flectens.* After its phylogenetic position was reexamined, *Pseudomonas flectens* was transferred to the family *Enterobacteriaceae* as *Phaseolibacter flectens* gen. nov., comb. nov. Here we describe the features of this organism, together with the draft genome sequence and annotation. The DNA GC content is 44.34 mol%. The chromosome length is 2,748,442 bp. It encodes 2,437 proteins and 89 RNA genes. *Ph. flectens* genome is part of the Genomic Encyclopedia of Type Strains, Phase I: the one thousand microbial genomes study.

**Electronic supplementary material:**

The online version of this article (doi:10.1186/s40793-015-0127-5) contains supplementary material, which is available to authorized users.

## Introduction

*Phaseolibacter flectens*ATCC 12775^T^ (= CFBP 3281^T^, ICMP 745^T^, LMG 2187^T^, NCPPB 539^T^), was isolated from infected French bean (*Phaseolus vulgaris*) pods in Queensland, Australia by Johnson (1956) [1]. Johnson, identified strain ATCC 12775^T^ as *Pseudomonas flectens* [1], however, 29 years later, De Vos et al. [2] argued, that *Ps. flectens* Johnson (1956) does not belong to the genus *Pseudomonas* and thus should be removed from this genus. Anzai et al. [3] demonstrated that *Ps. flectens* should be included in the cluster of the *Enterobacteriaceae* family [4]. Recently, Halpern et al. [5], reclassified the species *Ps. flectens* Johnson 1956 as the type species of a novel genus *Phaseolibacter* in the family *Enterobacteriaceae**,* as *Phaseolibacter flectens* gen. nov., comb. nov. [5]. Currently, the *Enterobacteriaceae* family comprises more than 60 different genera. Species belonging to this family exist in diverse environments such as water, terrestrial habitats, human, animals, insects or plants [4].

Johnson [1], studied a disease which caused blighting and twisting of French bean pods. He isolated strain ATCC 12775^T^ along with other strains that he identified as the same species from the diseased plants and proved that by inoculating healthy bean pods with pure culture of strain ATCC 12775^T^, the pods became twisted. The fact that the infection with *Ph. flectens* was confined to the pods, suggested that the introduction of the bacteria to the crop, took place after the flowering [1]. Johnson [1] demonstrated in experiments that were carried out in the laboratory and in a glasshouse, that bean thrips (*Taeniothrips nigricornis*), which are tiny, slender insects that feed on pollen and floral tissue, transmitted this plant pathogenic bacterium between the crop plants [1].

Here we describe a summary classification and a set of the features of the plant pathogenic bacterium *Ph. flectens*, together with the permanent draft genome sequence description and annotation of the type strain (ATCC 12775^T^).

## Organism information

### Classification and features

**Fig. 1:**
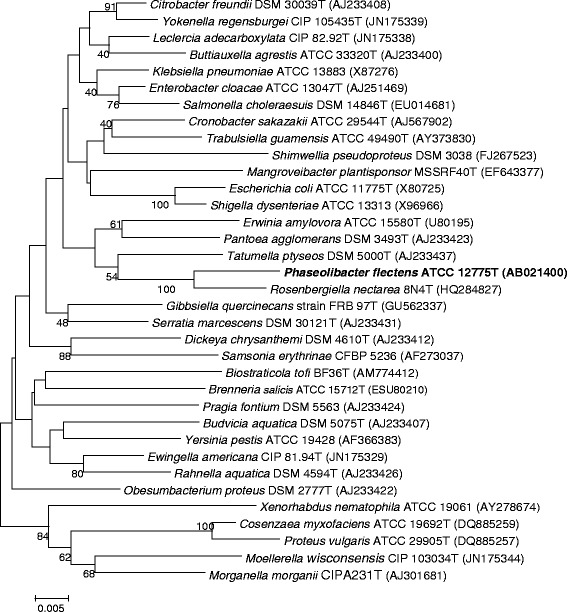
Phylogenetic tree highlighting the position of *Phaseolibacter flectens* relative to type species within the family *Enterobacteriaceae.* The sequence alignments were performed by using the CLUSTAL W program and the tree was generated using the neighbor joining method in MEGA 5 software [23]. Bootstrap values (from 1,000 replicates) greater than 40 % are shown at the branch points. The bar indicates a 0.5 % sequence divergence

*Ph. flectens* strain ATCC 12775^T^ share typical characteristics of *Enterobacteriaceae* members such as: Gram negative, facultative anaerobic, chemoheterotrophic rod, positive for catalase and glucose fermentation and negative for oxidase [5] (Table 1). The phylogenetic tree based on the 16S rRNA also supports the fact that strain ATCC 12775^T^ is a member of the family *Enterobacteriaceae* (Fig. 1), as was already suggested by Anzai et al. [3]. *Ph. flectens* is the type species of the genus *Phaseolibacter**,* which currently comprises only one species [5].

**Table 1:** Classification and general features of *Phaseolibacter flectens* strain ATCC 12775^T^ according to the MIGS recommendations [24], published by the genome standards consortium [25] and the names for life database [26]

MIGS ID	Property	Term	Evidence code^a^
	Current classification	Domain *Bacteria*	TAS [27]
		Phylum *Proteobacteria*	TAS [28]
		Class *Gammaproteobacteria*	TAS [29, 30]
		Order ‘*Enterobacteriales’*	TAS [31]
		Family *Enterobacteriaceae*	TAS [4]
		Genus *Phaseolibacter*	TAS [5]
		Species *Phaseolibacter flectens*	TAS [5]
		Type strain ATCC 12775^T^	TAS [1]
	Gram stain	Negative	TAS [1, 5]
	Cell shape	Rod	TAS [1, 5]
	Motility	Motile	TAS [1, 5]
	Sporulation	Non-sporulating	IDS
	Temperature range	4–44 °C	TAS [5]
	Optimum temperature	28–30 °C	TAS [5]
	pH range, optimum	Unknown	NAS
	Carbon source	Glucose	TAS [5]
MIGS-6	Habitat	Pods of French bean	TAS [5]
MIGS-6.3	Salinity	Unknown	NAS
MIGS-22	Oxygen requirement	Facultative anaerobic	TAS [5]
MIGS-15	Biotic relationship	Plant host-associated	TAS [5]
MIGS-14	Pathogenicity	Plant pathogen	TAS [1]
MIGS-4	Geographic location	Australia, Queensland	TAS [1]
MIGS-5	Sample collection	1956	TAS [1]
MIGS-4.1	Latitude	Unknown	NAS
MIGS-4.2	Longitude	Unknown	NAS
MIGS-4.4	Altitude	Unknown	NAS

^a^Evidence codes - *IDA* Inferred from Direct Assay, *TAS* Traceable Author Statement (i.e., a direct report exists in the literature); *NAS* Non-traceable Author Statement (i.e., not directly observed for the living, isolated sample, but based on a generally accepted property for the species, or anecdotal evidence). Evidence codes are from the Gene Ontology project [32]

**Fig. 2:**
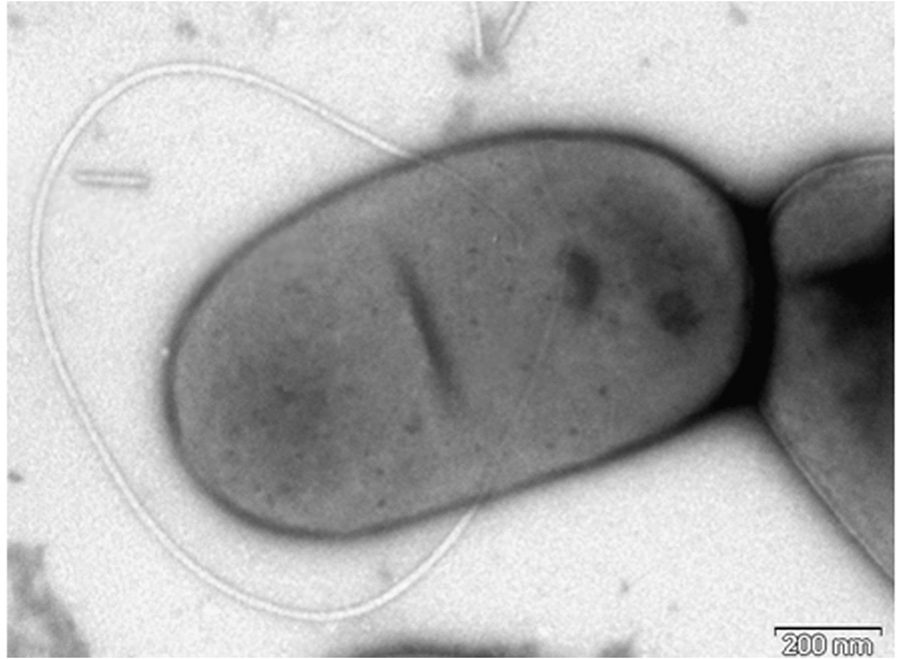
Electron micrograph of negatively stained cells of strain *Phaseolibacter flectens*. Cells are nonflagellated rods when grown on media supplemented with sucrose. However, a flagellum can be seen when the strain is grown on media without the supplementation of sucrose. Bar, 200 nm

Cells of *Ph. flectens* strain ATCC 12775^T^ are motile rods by means of one or two flagella, measuring 0.5–0.8 μm in width and 1.2–2.3 μm in length (Fig. 2). When cells are grown on LB or R2A agar media for 48 h, colonies are 1 mm diameter, however, when cells are grown on the same media supplemented with sucrose, colonies are 3–5 mm diameter, produce huge amount of mucus, smooth, foggy and grayish white colored and motility is not observed. Growth is observed under anaerobic conditions [5]. Grows at 4–44 °C (optimum, 28–30 °C), with 0–60 % sucrose (optimum, 10–25 % sucrose) (Table 1). Growth is observed on MacConkey agar. D-glucose, sucrose, D-melibiose, glycerol, D-fructose are fermented; acetoin is produced; H_2_S and indole are not produced; gelatin and urea are not hydrolyzed; citrate is not utilized; nitrate is reduced to nitrogen. L-arabinose, D-manitol, inositol, sorbitol, rhamnose, and amygdalin are not fermented. Tryptophane deaminase activity is present; ß-galactosidase, arginine dihydrolase, lysine and ornithine decarboxylases activities are absent [5].

#### Chemotaxonomic data

The major fatty acids are: C_16:0_; Summed feature 2 (one or more of C_14:0_ 3-OH, iso-C_16:1_ I and unknown ECL 10.928) and Summed feature 3 (C_16:1_*ω*7c and/or iso-C_15:0_ 2-OH) [2]. Minor fatty acids are: unknown 13.957; C_17:0_ cyclo; C_18:1_*ω*7*c*; C_12:0_; C_14:0_ 2-OH and C_14:0_ [5].

## Genome sequencing information

### Genome project history

This organism was selected for sequencing based on its phylogenetic position [6, 7] and is part of the study Genomic Encyclopedia of Type Strains, Phase I: the one thousand microbial genomes project [8]. The goal of the KMG-I study is to increase the coverage of sequenced reference microbial genomes [9]. The project is registered in the Genomes OnLine Database [10] and the permanent draft genome sequence is deposited in GenBank. Draft sequencing and assembly were performed at the DOE Joint Genome Institute (jgi.doe.gov) using state of the art sequencing technology [11]. A summary of the project information is shown in Table 2.

**Table 2:** Genome sequencing project information

MIGS ID	Property	Term
MIGS 31.1	Finishing quality	Level 2: high-quality draft
MIGS-28	Libraries used	Illumina std shotgun library
MIGS 29	Sequencing platforms	Illumina HiSeq 2000, Illumina HiSeq 2500
MIGS 31.2	Fold coverage	561X
MIGS 30	Assemblers	Velvet (v. 1.1.04), ALLPATHS–LG (v. r42328)
MIGS 32	Gene calling method	Prodigal 2.5
	Locus tag	L871
	Genbank ID	JAEE00000000
	Genbank date of release	23-JAN-2014
	GOLD ID	Gp0032039
	BIOPROJECT	PRJNA204094
MIGS-13	Source material identifier	ATCC 12775
	Project relevance	GEBA-KMG, tree of life

### Growth conditions and genomic DNA preparation

*Ph. flectens* strain ATCC 12775^T^ was grown in the appropriate medium as recommended on the web pages of the collection (Nutrient agar or broth). The purity of the culture was determined by growth on general maintenance media. Cells were harvested by centrifugation and genomic DNA was extracted from lysozyme-treated cells using cetyltrimethyl ammonium bromide and phenol-chloroform. The purity, quality and size of the bulk genomic DNA preparation was assessed according to DOE-JGI guidelines. Amplification and partial sequencing of the 16S rRNA gene confirmed the identity of strain 12775^T^.

### Genome sequencing and assembly

The draft genome of *Ph. flectens* was generated at the DOE Joint genome Institute (JGI) using the Illumina technology [12]. An Illumina std. shotgun library was constructed and sequenced using the Illumina HiSeq 2000 platform which generated 18,689,832 reads totaling 2,803.5 Mb. All general aspects of library construction and sequencing performed at the JGI can be found at the JGI website (jgi.doe.gov). All raw Illumina sequence data was passed through DUK, a filtering program developed at JGI, which removes known Illumina sequencing and library preparation artifacts (Mingkun L, Copeland A, Han J. DUK, unpublished, 2011). Following steps were then performed for assembly: (1). filtered Illumina reads were assembled using Velvet [13], (2). 1–3 kb simulated paired end reads were created from Velvet contigs using wgsim (https://github.com/lh3/wgsim), (3). Illumina reads were assembled with simulated read pairs using Allpaths–LG [14]. Parameters for assembly steps were: (1). Velvet (velveth: 63 –shortPaired and velvetg: −very clean yes –exportFiltered yes –min contig lgth 500 –scaffolding no –cov cutoff 10) (2). wgsim (−e 0 –1 100 –2 100 –r 0 –R 0 –X 0) (3). Allpaths–LG (PrepareAllpathsInputs: PHRED 64 = 1 PLOIDY = 1 FRAG COVERAGE = 125 JUMP COVERAGE = 25 LONG JUMP COV = 50, RunAllpathsLG: THREADS = 8 RUN = std shredpairs TARGETS = standard VAPI WARN ONLY = True OVERWRITE = True). The final draft assembly contained 29 contigs in 26 scaffolds, totalling 2.7 Mb in size. The final assembly was based on 1,500.0 MB of Illumina data.

### Genome annotation

The assembled sequence was annotated using the JGI prokaryotic annotation pipeline [15] and was further reviewed using the Integrated Microbial Genomes—Expert Review platform [16]. Genes were identified using Prodigal [17]. CRISPR elements were detected using CRT [18] and PILER-CR [19]. The final annotated genome is available from the Integrated Microbial Genome system [20].

## Genome properties

The assembly of the draft genome sequence consists of 26 scaffolds amounting to 2,748,442 bp, and the G + C content is 44.34 % (Table 3, Additional file 1: Table S1). Of the 2,526 genes predicted, 2,437 were protein-coding genes, and 89 RNAs. The majority of the protein-coding genes (81.2 %) were assigned a putative function while the remaining ones were annotated as hypothetical proteins. The distribution of genes into COGs functional categories is presented in Table 4.

**Table 3:** Genome statistics

Attribute	Value	% of Total
Genome size (bp)	2,748,442	100.00
DNA coding (bp)	2,272,995	82.70
DNA G + C (bp)	1,218,718	44.34
DNA scaffolds	26	100.00
Total genes	2,526	100.00
Protein coding genes	2,437	96.48
RNA genes	89	3.52
Pseudo genes	0	0.00
Genes in internal clusters	1,553	61.48
Genes with function prediction	2,051	81.20
Genes assigned to COGs	1,800	71.26
Genes with Pfam domains	2,103	83,25
Genes with signal peptides	179	7.09
Genes with transmembrane helices	552	21.85
CRISPR repeats	1

**Table 4:** Number of genes associated with the general COG functional categories

Code	Value	% age	Description
J	221	11.05	Translation, ribosomal structure and biogenesis
A	1	0.05	RNA processing and modification
K	104	5.20	Transcription
L	112	5.60	Replication, recombination and repair
B	0	0.00	Chromatin structure and dynamics
D	40	2.00	Cell cycle control, cell division, chromosome partitioning
V	44	2.20	Defense mechanisms
T	67	3.35	Signal transduction mechanisms
M	188	9.40	Cell wall/membrane biogenesis
N	34	1.70	Cell motility
U	69	3.45	Intracellular trafficking, secretion and vesicular transport
O	92	4.60	Posttranslational modification, protein turnover, chaperones
C	104	5.20	Energy production and conversion
G	104	5.20	Carbohydrate transport and metabolism
E	190	9.50	Amino acid transport and metabolism
F	65	3.25	Nucleotide transport and metabolism
H	112	5.60	Coenzyme transport and metabolism
I	75	3.75	Lipid transport and metabolism
P	104	5.20	Inorganic ion transport and metabolism
Q	38	1.90	Secondary metabolites biosynthesis, transport and catabolism
R	96	4.80	General function prediction only
S	95	4.75	Function unknown
-	726	28.74	Not in COGs

## Insights from the genome sequence

*Ph. flectens* was isolated from pods of diseased French bean plants. The genome of *Ph. flectens* strain ATCC 12775^T^ reveals the presence of virulence associated genes which demonstrate that indeed, this species has the potential to attack plant tissues. Salmonella-Shigella invasin protein C (IpaC SipC) gene is present in the genome of *Ph. flectens* and represents a family of proteins associated with bacterial type III secretion systems, which are injection machines for virulence factors into host cell cytoplasm. A heat labile enterotoxin alpha chain that belongs to the ADP-ribosylation superfamily, is also present in the *Ph. flectens* genome. Five genes in the genome of *Ph. flectens* encode the virulence factor hemolysin which has a lytic activity on eukaryotic cells. These genes are: hemolysin activation/secretion protein (two copies); hemolysin-coregulated protein; phospholipase/lecithinase/hemolysin; hemolysins and related proteins containing CBS domains and putative hemolysin. Two copies of a gene encoding filamentous hemagglutinin family N-terminal domain are encoded in the genome of strain ATCC 12775^T^, representing another virulence potential of this bacterium. Filamentous hemagglutinin-like adhesins are virulence factors in both plant and animal pathogens and have a role in the attachment, aggregation and cell killing [21]. Another feature of bacterial phytopathogenesis is the secretion of pectinolytic enzymes by microorganisms [22]. Pectate lyase (two copies) is found in the genome*,* demonstrating the potential of this species to degrade the pectic components of the plant cell wall.

The potential of *Ph. flectens* to produce pili is evident from the presence of seven pili genes: prepilin-type N-terminal cleavage/methylation domain; P pilus assembly protein, pilin FimA (eight copies); P pilus assembly protein, chaperone PapC (two copies); P pilus assembly protein, chaperone PapD (three copies); P pilus assembly/Cpx signaling pathway, periplasmic inhibitor/zinc-resistance associated protein; Type II secretory pathway, ATPase PulE/Tfp pilus assembly pathway, ATPase PilB and CblD like pilus biogenesis initiator (two copies).

The presence of the gene for S-ribosylhomocysteine lyase LuxS indicates that *Ph. flectens* produces quorum-sensing autoinducer 2 (AI-2).

## Conclusions

In the current study we characterized the genome of *Ph. flectens* strain ATCC 12775^T^, that was isolated from French bean pods in Queensland, Australia [1]. Strain ATCC 12775^T^ is a plant pathogen that cause pod twist disease in French bean plants. The bacteria cause the destruction of immature bean pods, immediately after the flowering stage. The blighted pods wither and drop to the ground or remain hanging and become twisted. Bean thrips (*Taeniothrips nigricornis*), are the ones that probably transmit this plant pathogenic bacterium between the crop plants [1]. Genes indicating the potential of strain ATCC 12775^T^ to cause plant disease were found in the bacterial genome. Among them were: injection machine for virulence factors into host cell cytoplasm (invasin protein C (IpaC_SipC)); heat labile enterotoxin; phospholipase/lecithinase/hemolysin which is capable of destroying the Eukaryotic cell membrane; filamentous hemagglutinin-like adhesins which have a role in the attachment, aggregation and host cell killing [21] and pectate lyase that has the potential to degrade the pectic components of the plant cell wall [22].

## Additional file

Additional file 1: Table S1. Scaffolds and contigs of Genomic DNA for *Phaseolibacter flectens* ATCC 12775^T^ (Topology; linear, Read depth; 1.00). (DOCX 24 kb)

## Abbreviations

KMG: One thousand microbial genomes
GEBA: Genomic encyclopedia of Bacteria and Archaea
MIGS: Minimum information about a genome sequence
TAS: Traceable
NAS: Non-traceable

## References

[1] Johnson JC (1956). Pod twist: a previously unrecorded bacterial disease of French bean (*Phaseolus vulgaris* L.). Qld J Agric Sci.

[2] De Vos P, Goor M, Gillis M, De Ley J (1985). Ribosomal ribonucleic acid cistron similarities of phytopathogenic *Pseudomonas* species. Int J Syst Bacteriol..

[3] Anzai Y, Kim H, Park JY, Wakabayashi H, Oyaizu H (2000). Phylogenetic affiliation of the pseudomonads based on 16S rRNA sequence. Int J Syst Bacteriol..

[4] Octavia S, Lan R, Rosenberg E, DeLong EF, Lory S, Stackebrandt E, Thompson F (2014). The Family *Enterobacteriaceae*. *The Prokaryotes* - *Gammaproteobacteria*.

[5] Halpern M, Fridman S, Aizenberg-Gershtein Y, Izhaki I (2013). Transfer of *Pseudomonas flectens* Johnson 1956 to *Phaseolibacter* gen. nov., in the family *Enterobacteriaceae*, as *Phaseolibacter flectens* gen. nov., comb. nov. Int J Syst Evol Microbiol.

[6] Wu D, Hugenholtz P, Mavromatis K, Pukall R, Dalin E, Ivanova NN (2009). A phylogeny-driven Genomic Encyclopaedia of Bacteria and Archaea. Nature..

[7] Göker M, Klenk HP (2013). Phylogeny-driven target selection for large-scale genome-sequencing (and other) projects. Stand Genomic Sci..

[8] Kyrpides NC, Woyke T, Eisen JA, Garrity G, Lilburn TG, Beck BJ (2013). Genomic encyclopedia of type strains, phase I: the one thousand microbial genomes (KMG-I) project. Stand Genomic Sci..

[9] Kyrpides NC, Hugenholtz P, Eisen JA, Woyke T, Göker M, Parker CT (2014). Genomic Encyclopedia of Bacteria and Archaea: sequencing a myriad of type strains. PLoS Biol..

[10] Reddy TBK, Thomas AD, Stamatis D, Bertsch J, Isbandi M, Jansson J (2015). The Genomes OnLine Database (GOLD) v. 5: a metadata management system based on a four level (meta)genome project classification. Nucleic Acids Res.

[11] Mavromatis K, Land ML, Brettin TS, Quest DJ, Copeland A, Clum A (2012). The fast changing landscape of sequencing technologies and their impact on microbial assemblies and annotations. PLoS ONE..

[12] Bennett S (2004). Solexa Ltd.. Pharmacogenomics.

[13] Zerbino DR, Birney E (2008). Velvet: algorithms for de novo short read assembly using de Bruijn graphs. Genome Res..

[14] Gnerre S, MacCallum I (2011). High–quality draft assemblies of mammalian genomes from massively parallel sequence data. Proc Natl Acad Sci USA.

[15] Mavromatis K, Ivanova NN, Chen IM, Szeto E, Markowitz VM, Kyrpides NC (2009). The DOE-JGI standard operating procedure for the annotations of microbial genomes. Stand Genomic Sci..

[16] Markowitz VM, Ivanova NN, Chen IMA, Chu K, Kyrpides NC (2009). IMG ER: a system for microbial genome annotation expert review and curation. Bioinformatics..

[17] Hyatt D, Chen GL, Locascio PF, Land ML, Larimer FW, Hauser LJ (2010). Prodigal: prokaryotic gene recognition and translation initiation site identification. BMC Bioinformatics..

[18] Bland C, Ramsey TL, Sabree F, Lowe M, Brown K, Kyrpides NC (2007). CRISPR recognition tool (CRT): a tool for automatic detection of clustered regularly interspaced palindromic repeats. BMC Bioinformatics..

[19] Edgar RC (2007). PILER-CR: fast and accurate identification of CRISPR repeats. BMC Bioinformatics..

[20] Markowitz VM, Chen I-M A, Palaniappan K, Chu K, Szeto E, Grechkin Y (2012). IMG: the integrated microbial genomes database and comparative analysis system. Nucleic Acids Res..

[21] Rojas CM, Ham JH, Deng WL, Doyle JJ, Collmer A (2002). HecA, a member of a class of adhesins produced by diverse pathogenic bacteria, contributes to the attachment, aggregation, epidermal cell killing, and virulence phenotypes of Erwinia chrysanthemi EC16 on Nicotiana clevelandii seedlings. Proc Natl Acad Sci USA.

[22] Mayans O, Scott M, Connerton I, Gravesen T, Benen J, Visser J (1977). Two crystal structures of pectin lyase A from Aspergillus reveal a pH driven conformational change and striking divergence in the substrate-binding clefts of pectin and pectate lyases. Structure..

[23] Tamura K, Peterson D, Peterson N, Stecher G, Nei M, Kumar S (2011). MEGA5: Molecular Evolutionary Genetics Analysis using Maximum Likelihood, Evolutionary Distance, and Maximum Parsimony Methods. Mol Biol Evol..

[24] Field D, Garrity GM, Gray T, Morrison N, Selengut J, Sterk P (2008). The minimum information about a genome sequence (MIGS) specification. Nat Biotechnol..

[25] Field D, Amaral-Zettler L, Cochrane G, Cole JR, Dawyndt P, Garrity GM (2011). The Genomic Standards Consortium. PLoS Biol..

[26] Garrity GM (2010). Names for Life Browser Tool takes expertise out of the database and puts it right in the browser. Microbiol Today..

[27] Woese CR, Kandler O, Wheelis ML (1990). Towards a natural system of organisms: proposal for the domains Archaea, Bacteria, and Eucarya. Proc Natl Acad Sci USA.

[28] Garrity GMBJ, Lilburn T, Garrity GM, Brenner DJ, Krieg NR, Staley JT (2005). Phylum XIV. *Proteobacteria* phyl. nov. Bergey's Manual of Systematic Bacteriology, Second Edition. 2 Part B.

[29] Garrity A (2005). Validation of publication of new names and new combinations previously effectively published outside the IJSEM. Int J Syst Evol Microbiol.

[30] Garrity GM, Holt JG, Lilburn T, Brenner DJ, Krieg NR, Staley JT, Garrity GM (2005). Class III. *Gammaproteobacteria* class. nov. Bergey’s Manual of Systematic Bacteriology, Second Edition.

[31] Garrity GMHJ, Garrity GM, Boone DR, Castenholz RW (2001). Taxonomic Outline of the Archaea and Bacteria. Bergey's Manual of System-atic Bacteriology.

[32] Ashburner M, Ball CA, Blake JA, Botstein D, Butler H, Cherry JM (2000). Gene ontology: tool for the unification of biology. The Gene Ontology Consortium. Nat Genet..

