# Supplementary material for: High quality permanent draft genome sequence of Phaseolibacter flectens ATCC 12775T, a plant pathogen of French bean pods
Source: Stand Genomic Sci. 2016 Jan 13;11:4. doi: 10.1186/s40793-015-0127-5 (PMC4710985; doi:10.1186/s40793-015-0127-5)
Supplement: Additional file 1: Table S1. — Scaffolds and contigs of Genomic DNA for Phaseolibacter flectens ATCC 12775T (Topology; linear, Read depth; 1.00). (DOCX 24 kb) [file 40793_2015_127_MOESM1_ESM.docx]

**Additional file 1**

**High quality permanent draft genome sequence of** [***Phaseolibacter flectens***](http://dx.doi.org/10.1601/nm.23736) [**ATCC 12775^T^**](http://doi.org/10.1601/strainfinder?urlappend=%3Fid%3DATCC+12775T)**, a plant pathogen of French bean pods**

Yana Aizenberg-Gershtein^1^, Ido Izhaki^1^, [Alla Lapidus](http://orcid.org/0000-0003-0427-8731)^2,3^, [Alex Copeland](http://orcid.org/0000-0002-3971-5439)^4^, TBK Reddy^4^, [Marcel Huntemann](http://orcid.org/0000-0002-1284-3748)^4^, Manoj Pillay^5^, Victor Markowitz^5^, [Markus Göker](http://orcid.org/0000-0002-5144-6200)^6^ Tanja Woyke^4^, [Hans-Peter Klenk](http://orcid.org/0000-0001-6758-8150)^7^, [Nikos C. Kyrpides](http://orcid.org/0000-0002-6131-0462)^4,8^ and Malka Halpern^1,9^*****

^1^Dept. of Evolutionary and Environmental Biology, Faculty of Natural Sciences, University of Haifa, Haifa, Israel

^2^Centre for Algorithmic Biotechnology, St. Petersburg State University, St. Petersburg, Russia.

^3^Algorithmic Biology Lab, St. Petersburg Academic University, St. Petersburg,

Russia

^4^ Dept. of Energy Joint Genome Institute, Genome Biology Program, Walnut Creek, CA, USA

^5^Biological Data Management and Technology Center, Lawrence Berkeley National Laboratory, Berkeley, CA, USA

^6^Leibniz Institute DSMZ—German Collection of Microorganisms and Cell Cultures, Braunschweig, Germany

^7^School of Biology, Newcastle University, Newcastle upon Tyne, UK

^8^ Dept. of Biological Sciences, Faculty of Science, King Abdulaziz University, Jeddah, Saudi Arabia

^9^ Dept. of Biology and Environment, Faculty of Natural Sciences, University of Haifa, Oranim, Kiryat Tivon, Israel

**Table S1.** Scaffolds and contigs of Genomic DNA for *Phaseolibacter flectens* ATCC 12775^T^ (Topology; linear, Read depth; 1.00).

| **Scaffold** | **Length (bp)** | **GC** | [**no. Genes**](https://img.jgi.doe.gov/cgi-bin/m/yui-dt0-href-NoGenes) |
| --- | --- | --- | --- |
| 1. Pseudomonas flectens ATCC 12775 : L871DRAFT_scaffold00001.1 | 839490 | 0.45 | 782 |
| 1. Pseudomonas flectens ATCC 12775 : L871DRAFT_scaffold00002.2 | 834587 | 0.44 | 774 |
| 1. Pseudomonas flectens ATCC 12775 : L871DRAFT_scaffold00003.3 | 162988 | 0.44 | 147 |
| 1. Pseudomonas flectens ATCC 12775 : L871DRAFT_scaffold00004.4 | 156728 | 0.44 | 139 |
| 1. Pseudomonas flectens ATCC 12775 : L871DRAFT_scaffold00005.5 | 149984 | 0.45 | 142 |
| 1. Pseudomonas flectens ATCC 12775 : L871DRAFT_scaffold00006.6 | 114890 | 0.45 | 115 |
| 1. Pseudomonas flectens ATCC 12775 : L871DRAFT_scaffold00007.7 | 96061 | 0.44 | 64 |
| 1. Pseudomonas flectens ATCC 12775 : L871DRAFT_scaffold00008.8 | 80018 | 0.43 | 66 |
| 1. Pseudomonas flectens ATCC 12775 : L871DRAFT_scaffold00009.9 | 72256 | 0.45 | 67 |
| 1. Pseudomonas flectens ATCC 12775 : L871DRAFT_scaffold00010.10 | 56681 | 0.43 | 47 |
| 1. Pseudomonas flectens ATCC 12775 : L871DRAFT_scaffold00011.11 | 55368 | 0.45 | 50 |
| 1. Pseudomonas flectens ATCC 12775 : L871DRAFT_scaffold00012.12 | 45032 | 0.45 | 42 |
| 1. Pseudomonas flectens ATCC 12775 : L871DRAFT_scaffold00013.13 | 36873 | 0.44 | 49 |
| 1. Pseudomonas flectens ATCC 12775 : L871DRAFT_scaffold00014.14 | 21241 | 0.46 | 15 |
| 1. Pseudomonas flectens ATCC 12775 : L871DRAFT_scaffold00015.15 | 6185 | 0.39 | 6 |
| 1. Pseudomonas flectens ATCC 12775 : L871DRAFT_scaffold00016.16 | 5872 | 0.43 | 7 |
| 1. Pseudomonas flectens ATCC 12775 : L871DRAFT_scaffold00017.17 | 2228 | 0.39 | 3 |
| 1. Pseudomonas flectens ATCC 12775 : L871DRAFT_scaffold00018.18 | 1774 | 0.32 | 1 |
| 1. Pseudomonas flectens ATCC 12775 : L871DRAFT_scaffold00019.19 | 1360 | 0.53 | 1 |
| 1. Pseudomonas flectens ATCC 12775 : L871DRAFT_scaffold00020.20 | 1279 | 0.37 | 1 |
| 1. Pseudomonas flectens ATCC 12775 : L871DRAFT_scaffold00021.21 | 1114 | 0.37 | 3 |
| 1. Pseudomonas flectens ATCC 12775 : L871DRAFT_scaffold00022.22 | 1148 | 0.58 | 1 |
| 1. Pseudomonas flectens ATCC 12775 : L871DRAFT_scaffold00023.23 | 1106 | 0.32 | 1 |
| 1. Pseudomonas flectens ATCC 12775 : L871DRAFT_scaffold00024.24 | 1106 | 0.32 | 1 |
| 1. Pseudomonas flectens ATCC 12775 : L871DRAFT_scaffold00025.25 | 1062 | 0.52 | 1 |
| 1. Pseudomonas flectens ATCC 12775 : L871DRAFT_scaffold_1000 | 2011 | 0.51 | 1 |
